# Supplementary material for: Data trace as the scientific foundation for trusted metrological data: a review for future metrology direction
Source: PeerJ Comput Sci. 2025 Aug 14;11:e3106. doi: 10.7717/peerj-cs.3106 (PMC12453846; doi:10.7717/peerj-cs.3106)
Supplement: Supplemental Information 2 [file peerj-cs-11-3106-s002.docx]

**Identification of studies via databases and registers**

Records removed *before screening*:

Records removed for not falling within the 2021-2025 publication range (n = 1828)

Records identified from*:

Databases (n = 3765),

using the keywords: "digital evidence" or "anti-forensics" or "digital forensics".

**Identification**

Records excluded:

Not containing the keywords "detection," "traces," or "tampering" (n = 1735)

Records screened

(n = 1937)

Reports excluded:

Excluding patents (n = 25)

Other (n = 11)

Reports sought for retrieval

(n = 202)

**Screening**

Reports excluded:

Not related to the research topic (n = 109)

Reports assessed for eligibility

(n = 166)

Reports incorporated:

Reports not retrieved but highly relevant (n = 24)

Reports targeted for retrieval

(n = 57)

**Included**

Studies included in review

(n = 81)

Source: Page MJ, et al. BMJ 2021;372:n71. doi: 10.1136/bmj.n71.

This work is licensed under CC BY 4.0. To view a copy of this license, visit <https://creativecommons.org/licenses/by/4.0/>
